# Genetic modifications of critical regulators provide new insights into regulation modes of raw-starch-digesting enzyme expression in *Penicillium*

**DOI:** 10.1186/s13068-022-02162-6

**Published:** 2022-05-31

**Authors:** Shengfang Zhao, Boyu Xiang, Le Yang, Jie Chen, Cui Zhu, Yu Chen, Jun Cui, Shengbiao Hu, Yibo Hu

**Affiliations:** 1 State Key Laboratory of Developmental Biology of Freshwater Fish, Hunan Provincial Key Laboratory for Microbial Molecular Biology, College of Life Science, Hunan Normal University, Changsha, China; 2 Faculty of Science and Medicine, University of Fribourg, Chemin du Musée 5, 1700 Fribourg, Switzerland

**Keywords:** Regulation network, Raw-starch-digesting enzyme, *Penicillium oxalicum*, Amylase

## Abstract

**Background:**

Starch is a very abundant and renewable carbohydrate and an important feedstock for industrial applications. However, most starch-based products are not cost-efficient due to the high energy input needed in traditional enzymatic starch conversion processes. Raw-starch-digesting enzymes (RSDEs) from filamentous fungi have great commercial value in starch processing. However, the regulatory mechanisms associated with their production in filamentous fungi remain unknown.

**Results:**

In this study, we reported the novel finding that cellulolytic fungus *Penicillium oxalicum* 114-2 has broad RSDE activity. Four regulators, including the amylase transcription activator AmyR, the catabolite repression repressor CreA, the group III G protein α subunit PGA3, and the nonhistone chromosomal protein HepA, have been found to play a crucial regulatory role in RSDE expression. Enzymatic assays revealed that RSDE production significantly increased after the overexpression of AmyR and HepA, the deletion of CreA and the dominant activation of PGA3. RT-qPCR analysis demonstrated that there is a mutual regulation mode between the four regulators, and then formed a cascade regulation mechanism that is involved in RSDE expression. Comparative transcriptomic analysis between the wild-type strain and genetically engineered strains revealed differentially expressed genes that may mediate the RSDE expression.

**Conclusions:**

The four different types of regulators were systematically investigated and found to form a regulatory network controlling RSDE gene expression. Our results provide a new insight into the regulatory mechanism of fungal amylolytic enzyme expression and offer a theoretical basis to rationally improve the RSDE yield in the future.

**Supplementary Information:**

The online version contains supplementary material available at 10.1186/s13068-022-02162-6.

## Background

Starch is the major carbohydrate reserve polymer in a large variety of higher plants, such as maize, cassava, wheat, potato, and oat. It is a potential substrate for the production of sugars and liquid fuels and chemicals [1–3]. Due to the polycrystalline structure in native starch granules [4], enzymatic hydrolysation at a higher temperature is required to efficiently disrupt the native starch structure in starch-based industrial processes. Briefly, raw starch was sequentially gelatinized at approximately 100 °C, liquefied with thermophilic α-amylases at approximately 95 °C, and treated with glucoamylases at 50–60 °C [5, 6]. Therefore, the conventional conversion of starch to glucose is energy intensive and time-consuming, thus increasing the production cost of starch-based products. In particular, the large amount of energy consumed in starch processing is also inconsistent with the current global strategy of "carbon peaking" and "carbon neutrality".

To reduce the energy costs associated with traditional starch biorefineries, raw-starch-digesting enzymes (RSDEs) could directly degrade raw starch granules into oligosaccharides or glucose below the gelatinization temperature of starch [7]. It has potential applications in food processing and biofuel preparation [8]. The RSDEs derived from *Aspergillus* sp. and *Rhizopus* sp. and *Bacillus* sp. are the most widely used and possess the following advantages: low production cost, good consistency, small time and space required for production, and easy process modification and optimization [9]. Although RSDEs are mainly secreted by filamentous fungi in the natural environment, they have low yields, which have not yet met the quantitative and cost requirements for large-scale industrialization of raw starch biorefining. The optimization strategy regarding the modification of RSDEs is mainly focused on two aspects. One approach to reduce the cost of raw starch biorefining is mining and identification of novel RSDEs that hydrolyse high concentration raw starches with high efficiency at a lower temperature. Recently, different α-amylases with high specific raw starch hydrolysing activity have been identified and characterized from bacteria and fungi [5, 10–13]. Wei Fang et al. identified a novel raw starch-digesting α-amylase AmyZ1 derived from marine bacterium *Pontibacillus* sp. ZY, and the AmyZ1 could efficiently hydrolyse raw starches at a relatively low temperature [14]. Additionally, a novel raw starch-digesting glucoamylase, PoGA15A, from *P. oxalicum,* was heterologously expressed by *P. pastoris,* displaying a high capacity for raw starch degradation and remarkable stability [6]. On the other hand, the improvement of RSDE production by using biotechnology approaches is a feasible strategy. Compared to those traditional strategies, such as random mutagenesis and fermentation optimization in the pregenomic era [15], rational genetic engineering transformation was predominantly employed to improve the yield of RSDEs. Generally, the expression of fungal RSDE genes is strictly controlled by upstream regulators, particularly transcription factors (TFs) at the transcriptional level. Some TFs have been proven to be significantly related to amylolytic enzyme production, such as Zn(II)2Cys6 zinc finger protein AmyR in *Aspergillus* species [16, 17]. Recently, a comparative transcriptomics study on *P. oxalicum* HP7-1 in the presence of glucose and starch revealed five transcription factors exhibiting significant regulation in the production of RSDEs. Among them, a transcription factor containing a pair of SANT/Myb domains controls the expression of an important RSDE gene, POX01356/PoxGA15A, in *P. oxalicum* [18]*.* In addition to these regulators, the HMG-box protein PoxHmbB and the extracellular protease activator PrtT, were identified as RSDE repressors in *P. oxalicum* [19, 20]*.* However, their research is insufficient to elucidate the regulatory mechanism behind the RSDE gene expression. Therefore, to further reduce the cost of enzyme production, it is rational to breed fungal strains based on an understanding of the molecular mechanisms controlling RSDE gene expression.

Apparently, *P. oxalicum* exhibits good prospects for raw amylase production and application in recent years [6, 15, 18]. The filamentous fungus *P. oxalicum* 114-2 has received substantial attention due to its highly efficient apparatus for cellulolytic enzyme synthesis. It has been under investigation for more than 30 years in China [21]. According to the genome annotation and analysis, five amylolytic enzyme genes were found to encode Amy15A (PDE_09417), Amy15B (PDE_05527), Amy13A (PDE_01201), Amy13B (PDE_01021), and Agl31A (PDE_03966) in *P. oxalicum*. However, secretome analysis performed through LC–MS/MS showed that Amy15A and Amy13A, which occupied 28.85% and 10.95% of the total protein, respectively, were the two major amylolytic enzymes in the wild-type strain [21, 22]. Numerous reported studies have focused on the regulator network of cellulase expression in *P. oxalicum*114-2 over the years [23]*.* Regulators, such as the classic TFs AmyR and CreA, nonclassical TFs, including a casein kinase CK2, proteins heterotrimeric G protein, and nonhistone chromosomal protein, have been identified to be involved in cellulase expression pathways [24–26]. Although there is indirect evidence that the cellulase expression regulators may mediate amylolytic enzyme expression, their function in RSDE expression has not been systematically elaborated. In this study, we found that amylases secreted by the WT 114-2 strain on starch medium display efficient enzymatic activity against different raw starches. Moreover, four engineered strains were constructed by genetic modification of the amylase transcription activator AmyR, the catabolite repression repressor CreA, the group III G protein α subunit PGA3 and the nonhistone chromosomal protein HepA in *P. oxalicum*. By employing RT-qPCR and RNA-seq technologies, the regulatory network was revealed and provided deep insights into the RSDE expression mode of *P. oxalicum*. Additionally, the suggested mechanisms behind regulators of RSDE expression might be general properties in amylolytic fungi and broadly enable engineering strategies for the protein hyperproducers.

## Results

### Raw starch-digesting enzyme activity and substrate specificity

**Fig. 1:**
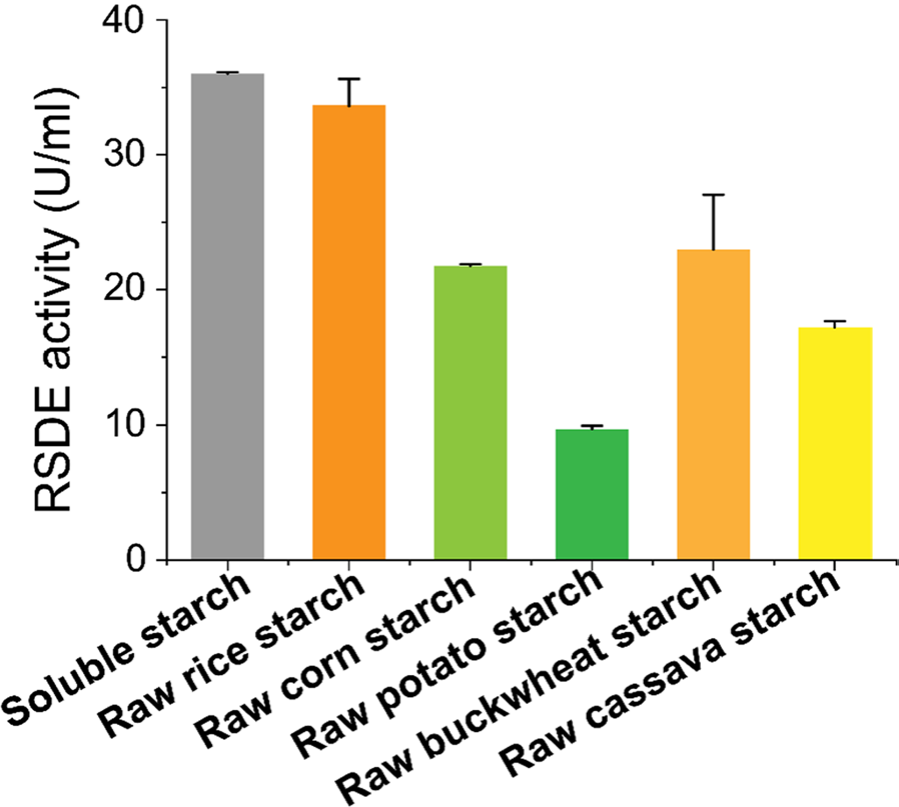
Enzyme activity of RSDEs towards soluble starch and various raw starches. The standard errors of the results with three replicates are indicated by error bars

To analyse the raw starch-digesting abilities of extracellular protein from WT 114-2, the strain was cultivated in Vogel’s minimal medium with starch as a sole carbon source and amylolytic enzyme expression inducer. Soluble starch and different raw starches derived from rice, corn, potato, buckwheat, and cassava were used as reaction substrates. The extracellular amylases showed different raw starch-digesting enzyme activities. Compared with soluble starch as a substrate, the extracellular enzymes exhibited higher RSDE activity towards raw rice starch, but lower RSDE activity towards the other four raw starches (Fig. 1). RSDEs secreted from the WT 114-2 strain preferentially hydrolysed raw rice starch, and the result is comparable to the single RSDE characteristic from the marine bacterium *Pontibacillus* sp. and *P. oxalicum* GXU20 [6, 14]. Then, the substrate specificity of RSDE towards raw starches was investigated using the abovementioned substrates. The results are shown in Table 1. RSDEs from the WT 114-2 strain had a broad range of substrate specificities, in particular, the activity of RSDEs against raw rice starch was almost equal to the activity against soluble starch as the enzymatic reaction substrate. It exhibited the highest specific activity of 451.4 ± 12.6 U/mg towards soluble starch. Its enzymatic activity towards rice raw starch (94.1%), were much higher than those towards tested raw starches, including corn (60.3%), potato (26.9%), buckwheat (62.5%), and cassava (47.7%).

**Table 1:** Substrate specificity of the extracellular RSDEs towards soluble starch and various raw starches

Substrate	Specific activity (U/mg extracellular protein)	Relative activity (%)
Soluble starch	451.4 ± 12.6	100.0 ± 0.5
Raw rice starch	424.9 ± 8.9	94.1 ± 4.2
Raw corn starch	272.1 ± 4.6	60.3 ± 1.0
Raw potato starch	121.2 ± 7.2	26.9 ± 1.6
Raw buckwheat starch	212.5 ± 28.6	62.5 ± 6.3
Raw cassava starch	215.3 ± 9.7	47.7 ± 2.1

The experiment was repeated three times and data are mean ± standard deviation from three replicates

### Scanning electron microscopy of raw rice starch hydrolysed by *P. oxalicum* RSDEs

**Fig. 2:**
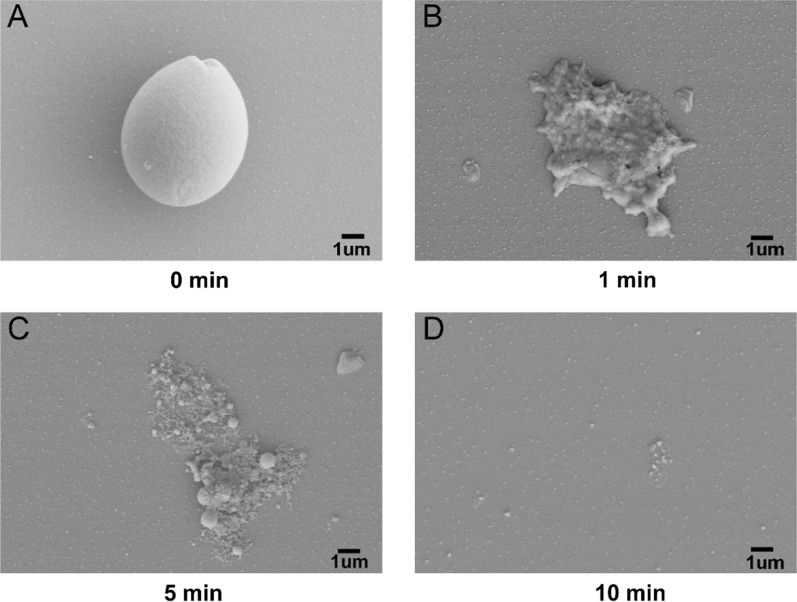
Scanning electron micrographs of starch granules digested by RSDEs. The raw rice starch granule treated after enzyme hydrolysis for 0 min (**A**), 1 min (**B**), 5 min (**C**), and 10 min (**D**)

Photographs of native and enzyme-hydrolysed starch were detected using scanning electron microscopy (SEM). Figure 2 shows that the surface of untreated raw rice starch granules was round and smooth (Fig. 2A). This shape is similar to the shape of raw cassava starch observed by SEM [6]. In contrast, when RSDEs attacked the starch, the shape of starch collapsed rapidly in 1 min (Fig. 2B), which was different from the observations of a large hole extending into the granule interior. With the extension of reaction time, most of the residual starch was further degraded within 5 min (Fig. 2C). Surprisingly, the residual starch granule almost disappeared after incubation with RSDEs for 10 min (Fig. 2D). This observation indicates that degradation of starch with mixed RSDEs is faster than that of a single enzyme, which displayed a hole-digging degradation pattern [6].

### Enhanced expression of RSDE production by genetic manipulation of four critic regulators

**Fig. 3:**
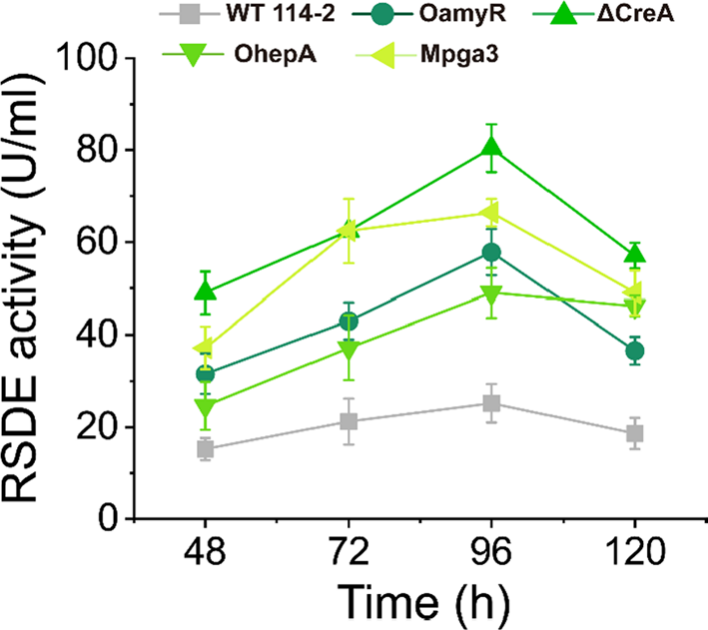
RSDE activity assay of WT and various mutants. The strains were cultivated in liquid VMM supplemented with 1% starch and cultivated at 30 °C for 5 days. Raw rice starch was used as the reaction substrate for RSDE activity assays

The reported studies and our primary research provided some clues that two TFs AmyR and CreA, a group III G protein α subunit PGA3 possibly mediate amylolytic enzyme expression [23, 25]. By employing genetic engineering techniques, we constructed a series of engineered mutant transformants including OamyR (*amyR* overexpressed under the *ubiD* strong constitutive promoter), ΔCreA (deletion of the *creA* gene), and Mpga3 (expressing a dominantly activated PGA3 with substitution of Gln208 by leucine), and OhepA (*hepA* overexpressed under the *ubiD* strong constitutive promoter). In particular, HepA was first identified for its role in regulating amylolytic enzyme expression in *P. oxalicum*. The four transformants were purified and identified by Southern blot and gene sequencing (Additional file 1: Fig. S1). The WT 114-2 and engineered strains were incubated in VMM plus soluble starch as a sole carbon source for 5 days. Expectedly, by using raw rice starch as a reaction substrate, markedly higher amylolytic enzyme activities were detected for both engineered strains compared with WT 114-2 during the fermentation period. The overexpression strains OamyR and OhepA showed 2.0- to 2.1-fold and 1.6- to 2.5-fold increases, respectively, compared with WT 114-2. On the fourth day, the RSDE activity of the CreA deletion strain ΔCreA reached 80.4 U/mL, which was 3.2-fold higher than that of WT 114-2. In particular, after dominant activation of the G protein PGA3, the level of RSDE activity increased by 2.4- to 2.9-fold in Mpga3 compared with the WT strain (Fig. 3). Moreover, amylolytic enzyme activity was further measured by using soluble starch as a reaction substrate, and the results were quite consistent with the results obtained by using raw rice starch as a reaction substrate (Additional file 1: Fig. S2). In addition, the HepA deletion strain (ΔhepA) and PGA3-deletion strain (ΔPGA3) were constructed in a preliminary study. Neither ΔHepA nor ΔPGA3 was measured with increased yield of RSDEs (Additional file 1: Fig. S3); therefore, the two transformants have not been further investigated.

**Fig. 4:**
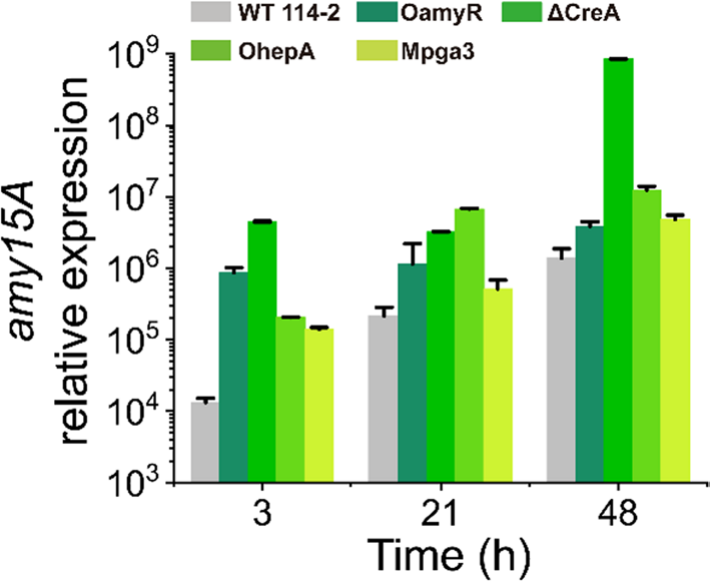
Expression levels of the *amy15A* gene in strains on raw rice starch. Relative transcription values represent the transcript copy numbers of each gene per 10^5^ copies of actin gene transcript. Error bars indicate the standard deviations of three independent measurements

To examine the transcriptional levels of RSDE genes in the four engineered strains, a RT-qPCR was carried out to compare the transcription patterns of the major RSDE gene *amy15A* [22]. As shown in Fig. 4, the transcript levels of *amy15A* in both engineered strains were obviously increased when compared with the WT strain. For OamyR, the transcript level of *amy15A* increased by 65.0-, 5.4- and 2.8-fold at 3, 21 and 48 h compared with the WT, respectively. The transcript levels of *amy15A* in OhepA were increased by 15.4-, 31.4- and 8.9-fold, respectively. The *amy15A* was upregulated remarkably in ΔCreA, and the expression of *amy15A* in ΔCreA was 5.2-, 2.8-, and 220.5-fold higher than that in WT at 3 h, 21 h and 48 h, respectively. The transcript abundance of *amy15A* was upregulated to 10.4-, 2.4-, and 3.5-fold in Mpga3 compared with that of the WT strain at the three detection points. The results suggested that overexpression of AmyR and HepA, deletion of CreA and dominant activation of PGA3 significantly activated RSDE gene expression at the transcript level.

### Influence on spore germination and hyphal extension of the mutant strains

**Fig. 5:**
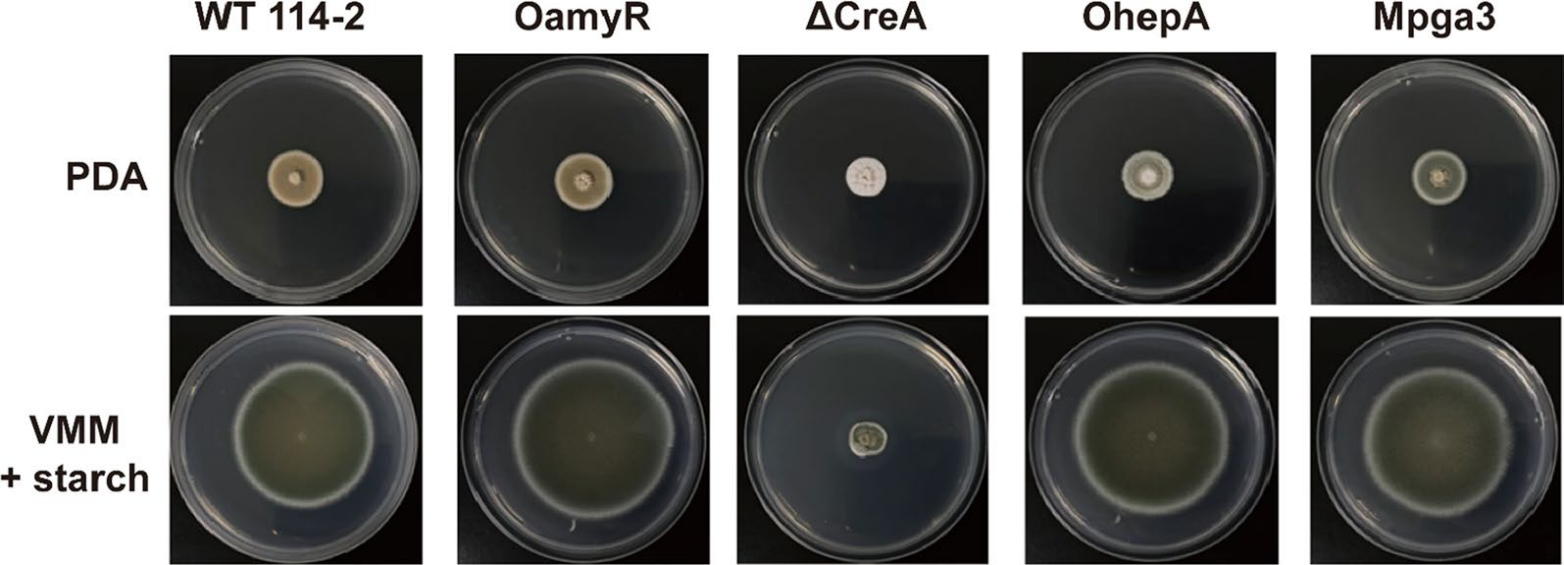
Colony morphology and conidiation of WT and mutant strains. Colony morphology of 6-day-old cultures for WT and mutant strains on PDA or VMM with 1% starch at 30 °C. The 1-μL conidial solutions of the WT and mutant strains were dropped onto agar at a density of 10^6^ conidia mL^−1^

To determine the effect of the genetic modifications on *P. oxalicum*, phenotypic analyses were conducted. Equal amounts of fresh spores collected from *P. oxalicum* 114-2, OamyR, ΔCreA, OhepA and Mpga3 were inoculated on solid-medium plates in the presence of starch as the sole carbon source and PDA solid medium, and cultured at 30 °C for 6 days. The colonies of OamyR and Mpga3 showed no obvious change when compared with the WT 114-2 strain on PDA and starch plates. The colony of ΔCreA on PDA was smaller than that of the WT strain. Since spore germination of *P. oxalicum* comes from white hyphae, the colonies gradually turn pale green with the development and maturation of spores. Therefore, we can infer that the deficiency of CreA would affect spore germination even in conditions with abundant nutrition (PDA medium) while restricting mycelium extension in nutrient limiting conditions (starch medium). Spores of the OhepA strain germinated and grew slower on PDA, while a similar phenotype on starch was observed compared with the WT strain (Fig. 5).

### Mutual regulation among the four regulators at the transcriptional level

**Fig. 6:**
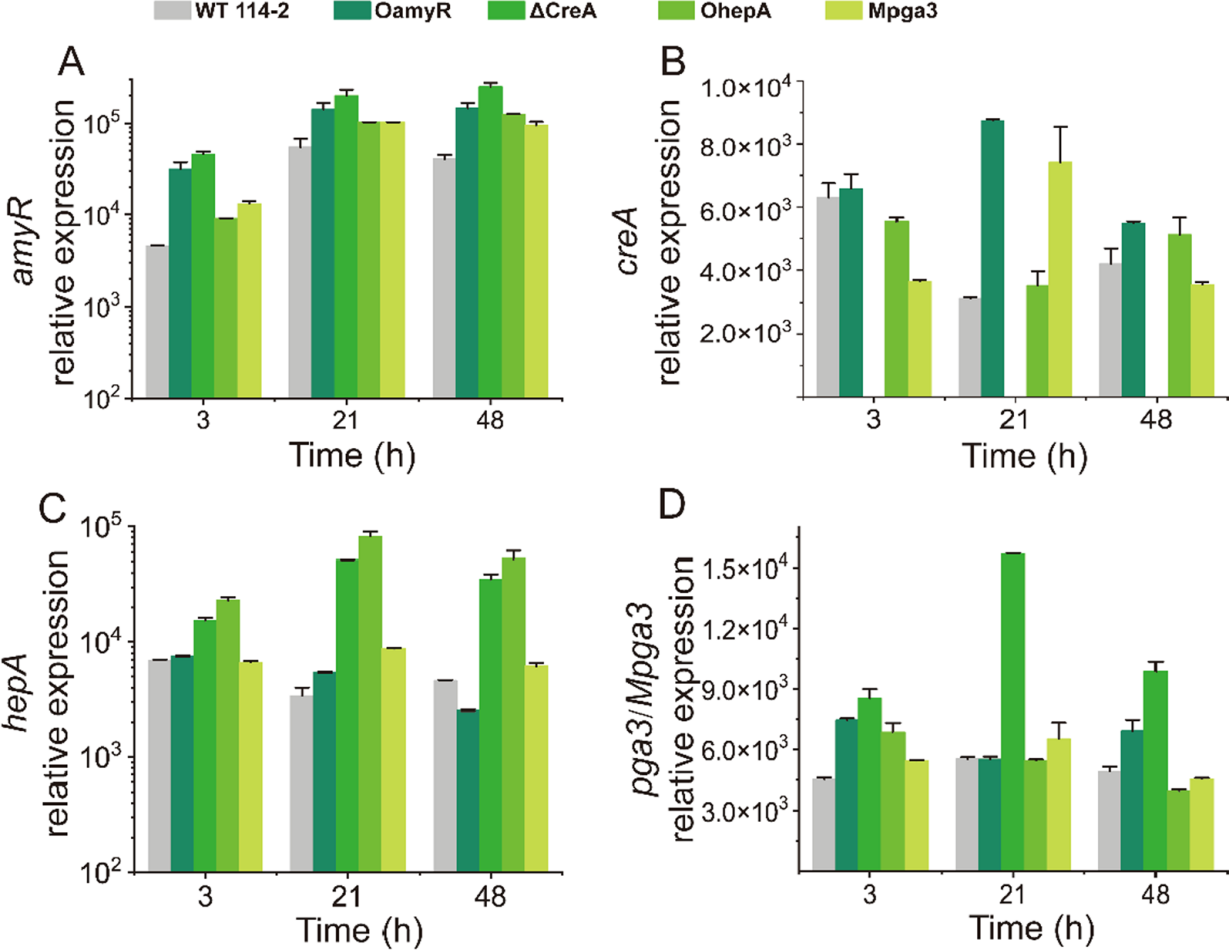
Transcription levels of *amyR* (**A**), *creA* (**B**), *hepA* (**C**), and *pga3/Mpga3* (**D**) in the WT and mutant strains. Relative transcription values represent the transcript copy numbers of each gene per 10^5^ copies of actin gene transcript. Error bars indicate the standard deviations of three independent measurements

Given the dose-controlled or additive regulation of glycoside hydrolases present in filamentous fungi [23, 25, 27], we assessed whether the four regulators, AmyR, CreA, HepA and PGA3 could affect each other’s expression at the transcriptional level by RT-qPCR detection. RT-qPCR was performed using total RNA from all strains grown on raw rice starch medium for 3 h, 21 h and 48 h after a shift from glucose. Four genes, *amyR*, *creA*, *hepA* and *pga3* (or *Mpga3*), were selected for evaluation in WT 114-2 and four engineered strains. As shown in Fig. 6, the expression of *amyR* on rice starch was upregulated to different extents in the four engineered strains relative to the WT (Fig. 6A), implying that the four regulators may activate RSDE gene expression by promoting *amyR* expression. The transcription level of the *creA* gene in OamyR was slightly higher, while no obvious trend could be summarized for its expression in the other three engineered strains (Fig. 6B). The results demonstrate that the transcript pattern of the carbon catabolite repressor gene *creA* was constitutive expression*.* The transcription of *hepA* and *pga3* was significantly upregulated in ΔCreA and exhibited no differential expression in the OamyR and Mpga3 strains (Fig. 6C and D). The results indicated that *pga3* and *hepA* gene expression was inhibited by carbon catabolite repressors in *P. oxalicum*.

### Transcription profiling of *P. oxalicum* 114-2 and the mutant strains

**Fig. 7:**
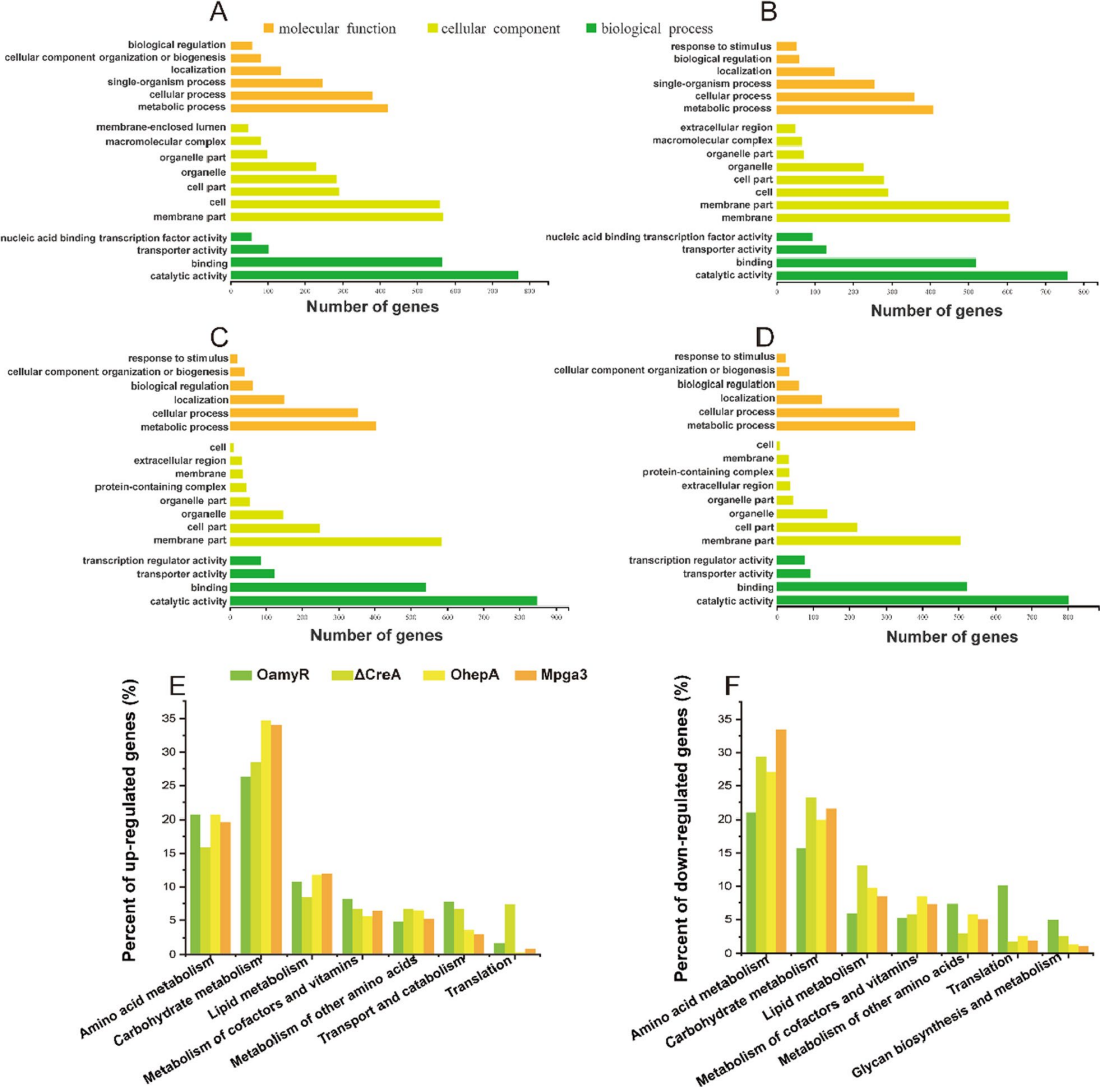
Comparative analysis of the transcriptomes of WT and mutant strains cultivated in starch medium. A GO enrichment analysis of differentially expressed genes (DEGs) in the OamyR (**A**), ΔCreA (**B**), OhepA (**C**), and Mpga3 (**D**) strains when compared with WT. Kyoto Encyclopedia of Genes and Genomes (KEGG) annotation of upregulated (**E**) and downregulated (**F**) genes in mutant strains when compared with WT

To further understand the regulatory mechanism of RSDE expression on starch, global gene expression changes in the WT, OamyR, ΔCreA, OhepA and Mpga3 strains were evaluated by RNA-Seq with three biological replicates. Total RNA was extracted from the mycelia of *P. oxalicum* grown on rice starch for 21 h and then sequenced. Every sample produced more than 50 million clean reads, with a < 0.025% error rate and > 92.5% mapped into the genome of the WT strain (Additional file 1: Table S1). The high consistency of samples among the three biological replicates made the transcriptome data more reliable. Transcriptome data showed that the expression levels of 1781, 1659, 1684, and 1832 genes exhibited significant differences (twofold or greater, FDR < 0.001) in the OamyR, ΔCreA, OhepA and Mpga3 strains when compared with WT 114-2 (data not shown). The GO terms that included the most abundant genes in molecular function, cellular component, and biological process were metabolic process (GO:0008152), membrane part (GO:0044425) and catalytic activity (GO:0003824), respectively (Fig. 7A–D). Furthermore, KEGG annotation indicated that upregulated and downregulated genes were most enriched in the process of amino acid metabolism and carbohydrate metabolism, demonstrating that synthesis of enzyme proteins and degradation of carbohydrates responded simultaneously in four engineered strains (Fig. 7E and F).

**Fig. 8:**
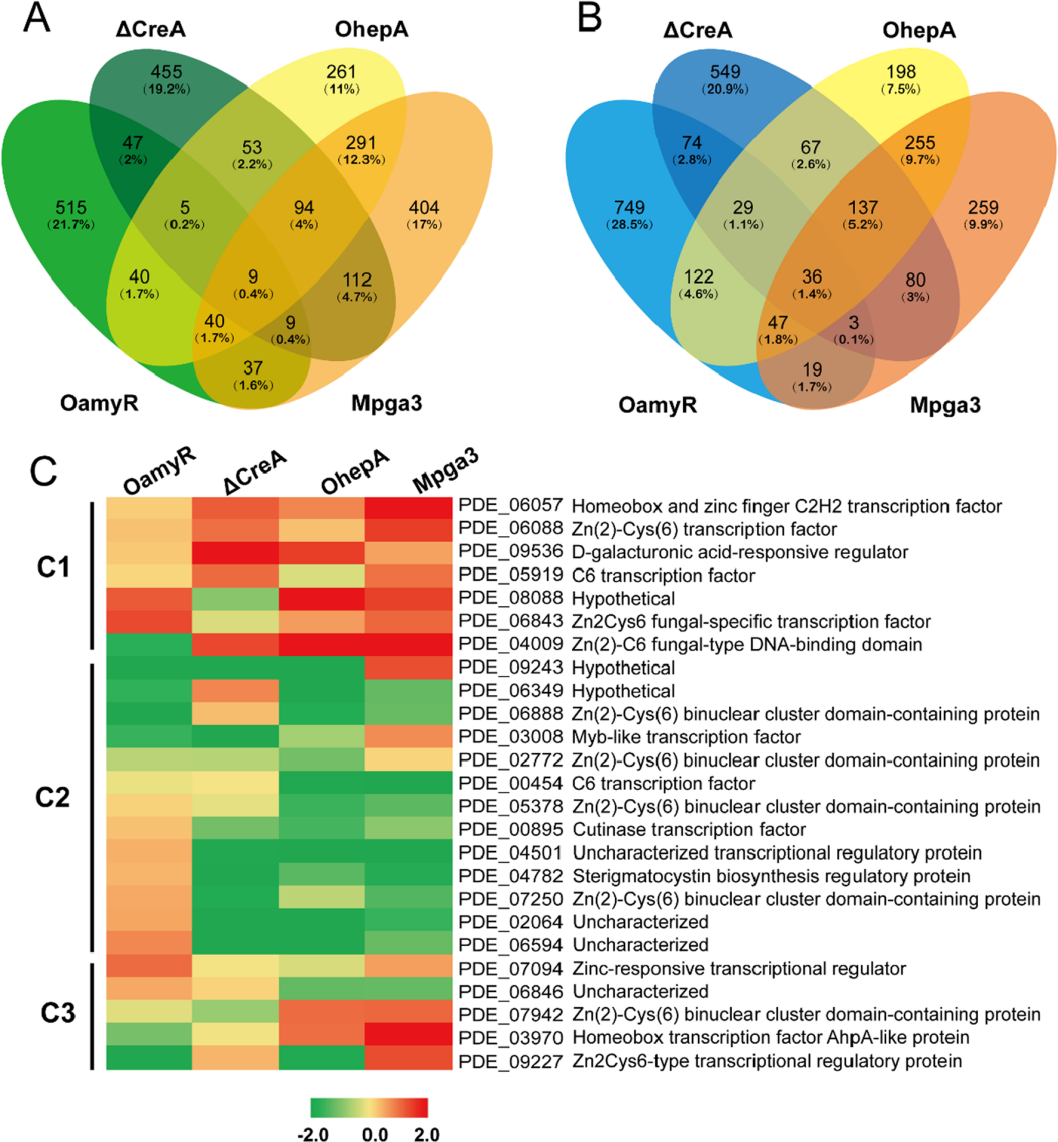
Venn diagram and heatmap analysis of differentially expressed genes in mutant strains when compared with WT. Venn diagram from the analysis of upregulated (**A**) and downregulated (**B**) genes in mutant strains when compared with WT. **C** Heatmap showing the transcription levels of DEGs encoding putative transcription factors in mutant strains. The transcription factor genes that were upregulated or downregulated simultaneously in two or more mutant strains are shown in this figure

Among the above DEGs, we were interested in the genes that were simultaneously upregulated and downregulated among the four engineered strains related to the genetic modification of RSDE regulators. Consequently, by using an FDR ≤ 0.01 and fold change ≥ 2 as the threshold, we determined that 702, 784, 793, and 996 genes were upregulated in response to the OamyR, ΔCreA, OhepA, and Mpga3 strain, respectively, compared with the WT 114-2 strain. Additionally, comparative transcriptome analyses identified 1079, 975, 891, and 836 downregulated genes in the four engineered strains compared with the WT strain, respectively. The Venn diagram shown indicates that 9 genes and 36 genes were found to be simultaneously upregulated and downregulated, respectively, in the four engineered strains (Fig. 8A and B). In particular, three amylolytic enzyme genes Amy15A (PDE_09417), Amy13A (PDE_01201) and Agl31A (PDE_03966), were found to be involved in the 9 co-upregulated genes (Table 2). Additionally, many genes annotated as uncharacterized and hypothetical proteins were included in those co-downregulated genes, demonstrating that those proteins may act as negative regulators of RSDE expression in *P. oxalicum* (Table 3).

**Table 2:** The commonly upregulated genes in four mutant strains

Gene ID	Accession number	NCBI annotation
PDE_09417	EPS34453	Glucoamylase Amy15A
PDE_07475	EPS32515	Cutinase
PDE_08879	EPS33917	Mitochondrial 2-oxoglutarate/malate carrier protein
PDE_01786	EPS26847	Aldehyde dehydrogenase
PDE_03966	EPS29020	Alpha-glucosidase
PDE_08974	EPS34012	SMP-30/gluconolactonase family protein
PDE_01577	EPS26639	Uncharacterized protein
PDE_04273	EPS29324	Chloroperoxidase domain-containing protein
PDE_01201	EPS26265	Alpha-amylase Amy13A

**Table 3:** The commonly downregulated genes in four mutant strains

Gene ID	Accession number	NCBI annotation
PDE_01187	EPS26251	Uncharacterized MFS-type transporter
PDE_09616	EPS34652	Alcohol dehydrogenase
PDE_01653	EPS26715	Uncharacterized protein
PDE_00509	EPS25575	Uncharacterized protein
PDE_08920	EPS33958	Calpain-type cysteine protease
PDE_06922	EPS31963	Major facilitator superfamily protein
PDE_09758	EPS34794	Uncharacterized protein
PDE_09761	EPS34797	Putative 4-hydroxyphenylpyruvate dioxygenase
PDE_06034	EPS31080	NPP1 domain protein
PDE_03291	EPS28345	Glucan endo-1,3-beta-d-glucosidase
PDE_06035	EPS31081	Putative *Podospora anserina* S mat genomic DNA chromosome 2
PDE_00508	EPS25574	Hypothetical protein
PDE_09759	EPS34795	Putative aromatic aminotransferase
PDE_03113	EPS28167	Ketoacyl-CoA thiolase
PDE_04812	EPS29862	MFS-type toxin efflux pump
PDE_08157	EPS33195	Hypothetical protein
PDE_04612	EPS29662	Hypothetical protein
PDE_06154	EPS31199	Hypothetical protein
PDE_02854	EPS27910	Uncharacterized protein
PDE_01193	EPS26257	Hypothetical protein
PDE_00518	EPS25584	Hypothetical protein
PDE_04129	EPS29180	Fungal Zn(2)-Cys(6) binuclear cluster domain-containing protein
PDE_01637	EPS26699	UDP-glucose 4-epimerase
PDE_09760	EPS34796	Hypothetical protein
PDE_01111	EPS26175	Uncharacterized protein
PDE_01192	EPS26256	Hypothetical protein
PDE_08014	EPS33052	Uncharacterized protein
PDE_04225	EPS29276	Fungal transcription factor
PDE_06571	EPS31616	Putative glutathione-dependent formaldehyde-activating enzyme
PDE_04614	EPS29664	Tyrosinase family protein
PDE_03290	EPS28344	Glycosyl transferase family 2 protein
PDE_04899	EPS29949	Uncharacterized protein PECM_007654
PDE_06706	EPS31749	Psi-producing oxygenase A
PDE_03776	EPS28830	Adhesin
PDE_07497	EPS32537	Bypass of stop codon protein 6
PDE_00144	EPS25212	Hypothetical protein

Generally, glycoside hydrolase expression is regulated by upstream transcription factor(s) in filamentous fungi [18, 23, 28]. TFs are considered essential players in signal transduction pathways, and are the last link between signal flow and target gene expression. Almost all putative transcription factor transcript levels in the four engineered strains were comparatively analysed with their transcript levels in WT 114-2 as a standard. We are interested in those transcription factors that are co-upregulated or co-downregulated in the four engineered strains. As a result, 25 transcription factors, most of which contained zinc-related structures (Zn_clus, C2H2, Zn2Cys6, etc.), were co-upregulated or co-downregulated in at least two or more mutants. As shown in Fig. 8C, Cluster 1 (C1) consisted of 7 TF genes displaying upregulation while C2 consisted of 14 TF genes displaying downregulation in three or four engineered strains. Among these 25 transcription factors, 5 TF genes (C3) were co-upregulated in the two mutants and co-downregulated in the other two strains. These data clearly indicated that the transcription of the putative TFs was regulated in different modes. In particular, of these genes, *PDE_06057* (Zn_clus), *PDE_06088* (Zn_clus) and *PDE_09536* (d-galacturonic acid-responsive regulator) were found to be co-upregulated, while only *PDE_02772* (Zn_clus) was found to be co-downregulated in the four engineered strains.

## Discussion

In the present study, we systematically investigated the degradation ability of *P. oxalicum* 114-2 on different raw starches. The results showed that the amylolytic enzymes secreted by the 114-2 strain had comprehensive degradation activity towards raw starch from different sources. The RSDEs from *P. oxalicum* 114-2 showed better amylolytic activity against rice raw starch, while they exhibited a faster deconstruction rate when degrading starch granules (Figs. 1 and 2). This may be due to the presence of other amylolytic enzymes, such as Amy13A, which may generate synergistic effects with Amy15A in the raw rice starch degradation process (Additional file 1: Fig. S4). The mixed RSDEs that mainly contained Amy15A and Amy13A showed higher activity against rice raw starch and lower activity against potato raw starch. The results are in agreement with those of PoGA15A from *P. oxalicum* GXU20 [6], demonstrating that the major RSDE Amy15A plays a dominant role in the degradation of raw starch. Unlike the heterologously expressed PoGA15A in *Pichia pastoris*, we obtained Amy15A by homologous expression in an engineered *P. oxalicum* host strain [29]. Although amino acid sequence alignment shows the high sequence identity between Amy15A and PoGA15A, some differences in enzymatic properties were found for the two amylolytic enzymes. Their pH stability was similar, but the temperature stability of the homologous-expressed Amy15A was not as high as that of the heterologous-expressed PoGA15A (Additional file 1: Figs. S5 and S6). This may be due to differences in posttranslational modifications in *Pichia pastoris*, which resulted in protein structure changes that were unexpectedly beneficial for enzyme temperature stability.

The expression of glycoside hydrolases in filamentous fungi is generally regulated by upstream regulators, with transcription factors being the most studied. Overexpression and knockdown of a single transcription factor are usually used to positively and negatively reveal the regulatory mechanism involved in glycoside hydrolase expression in filamentous fungi [30–32]. However, an increasing number of reports demonstrate that extracellular enzyme expression is not determined by a single regulator, but may be synergistic and dose-controlled by an upstream regulatory network consisting of multiple regulators [23, 27]. The four regulatory factors AmyR, CreA, HepA, and PGA3, which were previously confirmed as regulators involved in cellulase expression, were newly identified for their crucial regulatory function in RSDE expression in this study. Additionally, unlike those positive and negative studies of single transcription factors by application of gene overexpression/knockout technology, we tried to reveal the mode of RSDEs regulatory network from a positive insight by modifying the four regulators to make the downstream RSDEs production increased.

**Fig. 9:**
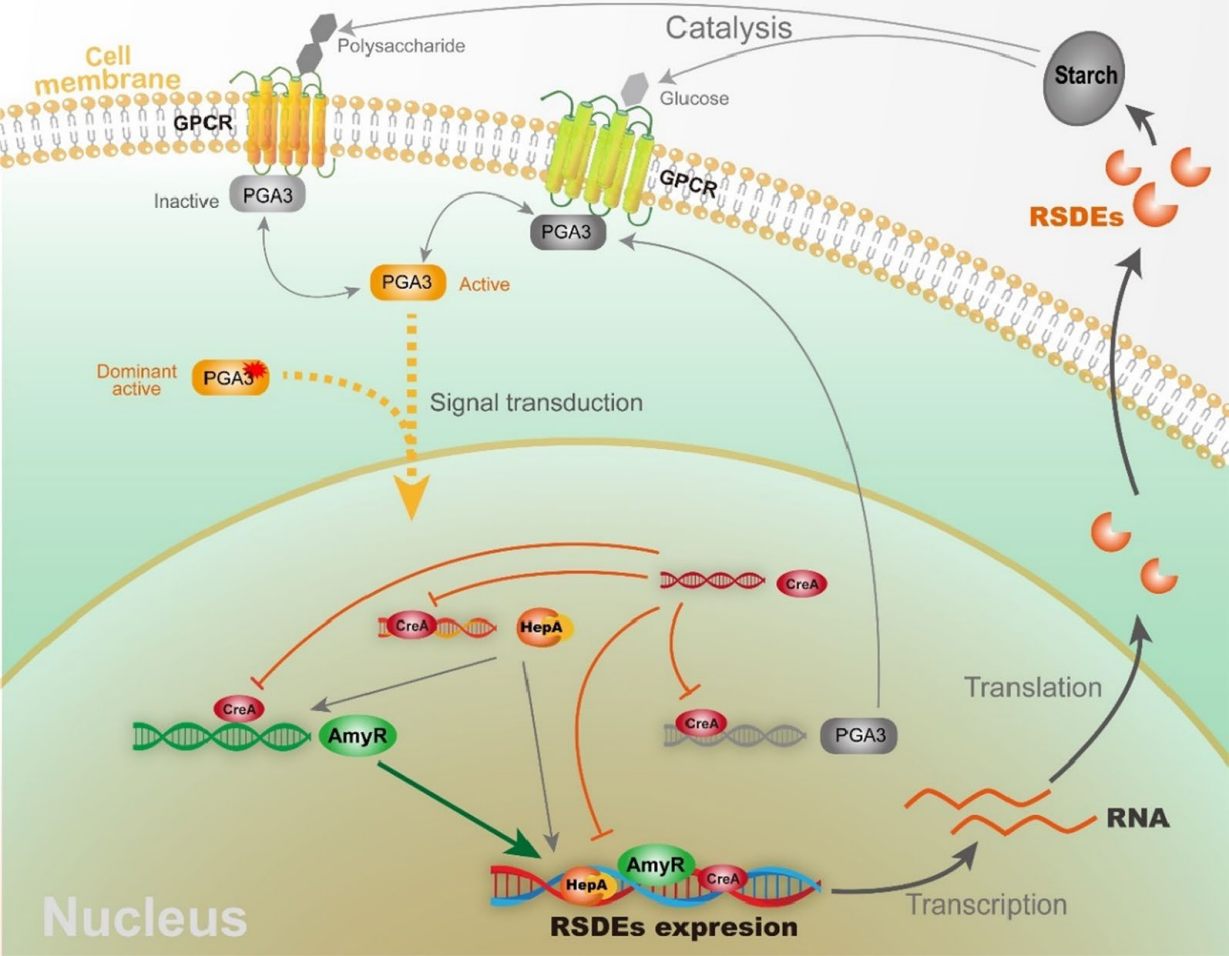
Schematic model of the role of the four regulators in regulating the production of RSDE enzymes in *P. oxalicum*. Solid lines represent possibly direct regulations, while dashed lines represent indirect regulations

We can speculate about the possible regulatory network model involved in RSDE expression. The plasma membrane-associated G protein binding of an extracellular ligand to transmembrane G protein-coupled receptor (GPCR) leads to GDP–GTP exchange on the Gα subunit and its activation [25, 33]. The Gα subunit PGA3 was dominantly activated and enhanced the expression of AmyR, which in turn increased the expression of RSDEs (Figs. 4 and 6A). Therefore, we can speculate that the G protein signalling pathway, a central signalling cascade with crucial functions in all organisms, may mediate the expression of RSDEs by transmitting external signals. CreA, as a homologue of the major carbon catabolite repressors in phylogenetically diverse fungi, played a negative role in RSDE expression [34, 35]. This regulator allows an organism to utilize a preferred carbon source but hinders it from metabolizing complex carbon sources, including raw starch. Deletion of CreA to relieve catabolite repression not only increased RSDE gene expression but also enhanced the expression of their upstream activators. It is suggested that CreA might have a cascade regulation because it repressed the activator genes of AmyR, PGA3 and HepA as well as the structural genes whose expression was upregulated by the three regulators (Fig. 6); therefore, the significant improvement of RSDE yield may be due to the “double-enhancement” effect after deleting CreA (Figs. 3 and 4). In addition, overexpression of HepA, a protein associated with a highly compact chromatin state [36, 37], may alter the structure of chromosomes to make it accessible for transcription machinery to attach to target genes. In particular, the regulators CreA and HepA are conserved in filamentous fungi [34–37]; therefore, the possible existence of an intimate crosstalk among certain developmental processes, such as sporulation and RSDE production pathways, is mediated by those regulators in ascomycete fungi (Fig. 5). AmyR, a master and key transcription factor, was exceptionally identified and positively regulated amylolytic enzyme gene expression in *P. oxalicum.* We speculated that the activation of RSDE expression by other regulators (i.e., CreA and HepA) may be partially due to enhanced AmyR expression (Fig. 6A). Taken together, in contrast to investigation of the regulation of multiple transcription factors, the expression of RSDE genes was found to be regulated by various global regulators in this study (Fig. 9). We further expanded our understanding of the regulatory network of RSDE expression by analysing regulators in different regulatory manners. Although comprehensive and intensive analyses are required, this study may provide a “stereoscopic” or possible temporal regulation mode for RSDE expression in *P. oxalicum*.

## Conclusions

While RSDE-assisted starch industrial processing has been a topic of study over the years, the gaps in our knowledge regarding the RSDE expression and the regulation of the pathways could be considered actual bottlenecks in the development of more effective genetic engineering strategies. In this study, we identified a number of common genes and metabolic pathways that mediate RSDE expression through global transcriptome analysis of four engineered strains. These common upregulated or downregulated genes are most likely to be involved in the regulation of RSDE expression, which will provide reduced but valuable targets for the next step of optimizing the regulatory network. These sophisticated regulatory mechanisms may also function to optimize carbon and energy flux. We will further investigate these common genes to explore more signalling transmitters or regulatory factors to improve the regulatory network of RSDE expression, thus providing theories for the most fruitful means of genetic modification to obtain higher industrial RSDE yield in the future.

## Methods

### Strains and culture conditions

The wild-type strain 114-2 was deposited at the China General Microbiological Culture Collection Center (CGMCC) under the number CGMCC 5302 and used throughout this work as a control. Raw starches (rice, corn, potato, buckwheat and cassava) were purchased from a local market in Changsha, China. The raw starch substrates used for the enzymatic activity assay were washed at least three times with ultra-pure water. Soluble starch was purchased from Sigma-Aldrich (St. Louis, MO, USA). The strains listed in Table 4 were routinely grown in wheat bran extract slants at 30 °C to obtain fresh conidia. For phenotypic analyses of strains on agar plates, Vogel's minimal medium (VMM) supplemented with 1% (wt/vol) soluble starch and PDA was used [38], and the conidia of strains were spotted in 1-μL aliquots. All strains were grown on solid plates at 30 °C for 6 days. For microscopic observation and RSDE activity determination, conidia of strains at a final concentration of 10^7^ per mL were inoculated into and grown in liquid glucose minimal medium for 24 h. Then, mycelia were collected by vacuum filtration, and 0.5 g wet mycelia were resuspended in 100 mL liquid VMM supplemented with 1% (wt/vol) soluble starch for further growth. The cultures were maintained at 30 °C on a rotary shaker at 200 rpm. The fermentation supernatant was used for microscopic observation of starch degradation and amylolytic enzyme activity determination.

**Table 4:** *P. oxalicum* strains used in this study

Strain name	Description	Parent strain	Reference
114-2	Wild type	–	[21]
OamyR	*ubiD*(p)::*amyR*-*hph*	WT 114-2	This study
OhepA	*ubiD*(p)::*hepA*-*hph*	WT 114-2	This study
ΔCreA	Δ*creA*-*hph*	WT 114-2	This study
Mpga3	*pga3*(p)::*Mpga3*-*hph*	WT 114-2	This study
ΔhepA	Δ*hepA*-*hph*	WT 114-2	This study
ΔPGA3	Δ*pga3*-*hph*	WT 114-2	This study

### Construction of targeting cassettes and transformation of *P. oxalicum*

The genomic DNA used as a PCR template was extracted from young hyphae grown on PDA liquid medium. The knockout or overexpression cassettes for each candidate gene were constructed by fusion PCR [39]. The cassettes contained a 1.9-kb *hph* gene and DNA fragments approximately 2.0 kb upstream and downstream of the target gene, and the DNA fragments were amplified by PCR using the corresponding primer pairs (Additional file 1: Table S2), For site-directed mutagenesis of *pga3*, the coding sequence of *Mpga3* (expressing a dominantly activated PGA3 with substitution of Gln208 by leucine), which contains a mutant site, was synthesized. The template of the *pga3* mutagenesis cassette was obtained by fusing the *Mpga3* coding region, *hph* cassette, and 3' flanking region (Additional file 1: Fig. S1). For all transformations of *P. oxalicum*, protoplasts were prepared based on a modified method as described by Gao et al. [40].

### Southern blot and sequencing

To determine the integration type of targeting cassettes in transformants, Southern blot analyses were performed (Additional file 1: Fig. S1). The mycelia were collected and genomic DNA was extracted as described previously [41]. The prepared genomic DNA (approximately 30 μg) of the OamyR, OhepA, ΔCreA, Mpga3 and 114-2 strains was digested by restriction enzymes, respectively, and then separated using agarose gel electrophoresis. Three probes were amplified for strain OamyR/OhepA, ΔCreA and Mpga3 analysis. A DIG-High Prime DNA Labelling and Detection Starter Kit I (Roche, Basel, Switzerland) was used for probe preparation, fragment hybridization, and immunological detection according to the manufacturer’s instructions. The coding region sequence of *Mpga3* was amplified from the Mpga3 transformants, and the mutations were confirmed by Sanger sequencing (BGI, Shenzhen, China).

### Enzyme assays and SDS-PAGE analysis

The raw starch-digesting enzyme activity was measured according to the DNS method [42]. The absorbance of the reaction system was measured at 540 nm. Up to 1.5 mL of starch solution was added for RSDE activity assays, and the reaction was incubated at 40 °C for 10 min. One unit of enzymatic activity (U) was defined as the amount of enzyme required to produce 1 μmol of reducing sugars per min from the reaction substrate. SDS-PAGE was performed using 12% polyacrylamide to determine protein purity. The protein profile was analysed by staining gels with Coomassie Brilliant Blue R-250 (Sangon Biotech, Shanghai, China) and destaining gels with 10% (w/v) acetate solution.

### RNA isolation, cDNA synthesis and quantitative RT-PCR

We validated the expression pattern of related genes by RT-qPCR. Total RNA was extracted using TRIzol reagent (TaKaRa, Dalian, China), and complementary DNA was generated using a reverse transcription kit following the manufacturer’s instructions. Quantitative real-time PCR was performed using SYBR Premix Ex Taq™ (Perfect Real Time) (TaKaRa, Dalian, China) with the primers listed in Additional file 1: Table S2. Each amplification reaction was in a total reaction volume of 20 µL. The thermal cycling protocol was as follows: initial denaturation for 2 min at 95 °C followed by 40 cycles of 10 s at 95 °C and 30 s at 61 °C. The fluorescence signal was measured at the end of each extension step at 80 °C. The reactions were performed in triplicate, and average transcription levels were determined and normalized to corresponding actin gene expression levels as an internal control.

### RNA-sequencing

Fresh spores of each strain were inoculated into 100 mL of 1% glucose medium and incubated at 200 rpm and 30 °C for 24 h. Equal amounts of mycelia were transferred to 1% raw rice starch medium and cultured for 21 h. Total RNA was extracted from the different samples by using a TRIzol total RNA extraction kit (TaKaRa, Dalian, China) and treated with DNase I (TaKara, Dalian, China) to remove the DNA. Transcriptome assays based on Illumina sequencing technology were performed at Shanghai Majorbio Biopharm Technology Co., Ltd (Shanghai, China). After quality control, the generated clean reads were mapped against predicted transcripts from the *P. oxalicum* 114-2 genome. Transcript abundance (fragments per kb per million reads, FPKM) genes with significantly different expression levels were identified through a significance test with combined thresholds (FDR ≤ 0.01 and fold change ≥ 2). Pearson’s correlation coefficient was used to evaluate transcriptome reliability, and three biological replicates were used in each sample. Differential gene expression was analysed by using the Majorbio cloud computing platform, included a series of DESeq software packages.

## Supplementary Information

**Additional file 1: Fig. S1.** Southern blot analysis of the genomic DNA of OamyR, OhepA, ΔCreA, and Mpga3 mutants. The location of the probes and restriction enzyme sites for Southern blot analysis are shown. The primers are listed in Table S2. (A) The overexpression strains OamyR and OhepA generated 5.6 and 4.7 kb DNA bands, respectively, while the parental strain 114–2 did not produce a detectable band, indicating that the overexpression cassettes were integrated into the genome. (B) Southern blot analysis of the genomic DNA of 114-2 and Mpga3. The 114-2 strain generated a 1.3 kb DNA band, while a 3.1 kb band was obtained in Mpga3 indicating that the targeted gene was correctly replaced. Sequencing of the mutated *pga3* (*Mpga3*) gene in strain Mpga3. The mutated codon is indicated. (C) Southern blot analysis of the genomic DNA of 114-2 and ΔCreA. The 114-2 strain generated a 3.3 kb DNA band, while a 6.3 kb band was obtained in ΔCreA indicating that the targeted gene was correctly replaced. **Fig. S2.** RSDE activity assay of WT and various mutants. The strains were cultivated in liquid VMM supplemented with 1% starch and cultivated at 30 °C for 5 days. Soluble starch was used as a reaction substrate for RSDE activity assays. **Fig. S3.** RSDE activity assay of WT, ΔHepA and ΔPGA3 strain. The strains were cultivated in liquid VMM supplemented with 1% starch and cultivated at 30 °C for 5 days. Raw rice starch was used as a reaction substrate for RSDE activity assays. **Fig. S4.** Extracellular proteins of 114-2 on starch analysed by SDS-PAGE. 32 μL culture supernatant of 114-2 strain was loaded after cultivating in 1% starch medium for 72 h, respectively. Lane M was the molecular weight marker, lanes 1 and 2 represent two independent cultivations for 114-2 strain. **Fig. S5.** SDS-PAGE analysis of the purified raw starch digesting enzyme Amy15A. Lane M indicates protein molecular weight marker; lane 1 indicates the purified Amy15A. **Fig. S6.** Effects of pH and temperature on enzymatic activity and the stability of the RSDE Amy15A. (A) The effect of pH on enzyme activity. The enzyme activity was assayed in a citrate–phosphate buffer (pH 3.0–7.0) at 37 °C. (B) The pH stability of Amy15A was measured by pre-incubating the enzyme in various buffers for 24 h at 4 °C, and the residual enzyme activity was determined using the standard method. (C) The influence of temperature on enzyme activity. The enzyme activity was determined between 35 °C and 80 °C under optimum pH condition. (D) The influence of temperature on enzyme stability. Temperature stability was determined by the standard method after pre-incubating the enzyme at pH 4.5 between 30 °C and 75 °C for 1 h. Data given are mean ± standard deviation from three replicates. The results are from a representative experiment, and similar results were obtained in two other independent experiments. **Table S1.** Quality control statistics. **Table S2.** Primers used in this study.

## Data Availability

All data generated or analysed during this study are included in this published article and its additional files.

## Abbreviations

RT-qPCR: A real-time quantitative polymerase chain reaction
WT: Wild type
TF: Transcription factor
LC–MS/MS: Liquid chromatography mass spectrometry
VMM: Vogel's minimal medium
DNS: 3,5-Dinitrosalicylic
SDS-PAGE: Sodium dodecyl sulfate–polyacrylamide gel electrophoresis
PDA: Potato dextrose agar
SEM: Scanning electron microscopy
DEGs: Differentially expressed genes
GO: Gene Ontology
KEGG: Kyoto Encyclopedia of Genes and Genomes
GPCR: G protein-coupled receptor
GDP: Guanosine 5'-diphosphate
GTP: Guanosine triphosphate
